# The free health care initiative: how has it affected health workers in Sierra Leone?

**DOI:** 10.1093/heapol/czv006

**Published:** 2015-03-21

**Authors:** Sophie Witter, Haja Wurie, Maria Paola Bertone

**Affiliations:** ^1^ReBUILD/IIHD, Queen Margaret University, Edinburgh, Scotland,; ^2^ReBUILD programme, College of Medicine and Allied Health Sciences, Freetown, Sierra Leone and; ^3^London School of Hygiene and Tropical Medicine, London, England

**Keywords:** Human resources for health, Sierra Leone, user fee removal

## Abstract

There is an acknowledged gap in the literature on the impact of fee exemption policies on health staff, and, conversely, the implications of staffing for fee exemption. This article draws from five research tools used to analyse changing health worker policies and incentives in post-war Sierra Leone to document the effects of the Free Health Care Initiative (FHCI) of 2010 on health workers.

Data were collected through document review (57 documents fully reviewed, published and grey); key informant interviews (23 with government, donors, NGO staff and consultants); analysis of human resource data held by the MoHS; in-depth interviews with health workers (23 doctors, nurses, mid-wives and community health officers); and a health worker survey (312 participants, including all main cadres). The article traces the HR reforms which were triggered by the FHCI and evidence of their effects, which include substantial increases in number and pay (particularly for higher cadres), as well as a reported reduction in absenteeism and attrition, and an increase (at least for some areas, where data is available) in outputs per health worker. The findings highlight how a flagship policy, combined with high profile support and financial and technical resources, can galvanize systemic changes. In this regard, the story of Sierra Leone differs from many countries introducing fee exemptions, where fee exemption has been a stand-alone programme, unconnected to wider health system reforms. The challenge will be sustaining the momentum and the attention to delivering results as the FHCI ceases to be an initiative and becomes just ‘business as normal’. The health system in Sierra Leone was fragile and conflict-affected prior to the FHCI and still faces significant challenges, both in human resources for health and more widely, as vividly evidenced by the current Ebola crisis.

**Key Messages**

- A health financing change, like the FHCI in Sierra Leone, can be a catalyst for broader health system reforms, given the right conditions (political and donor support and, perhaps, a sense of urgency resulting from a post-conflict situation).
- HRH reforms are at the core of making free health care function effectively. This article documents how Sierra Leone undertook some of the necessary changes to make health staff available and motivated to provide services at the time when the FHCI was introduced and after. These changes were introduced rapidly at first, and then with additional measures over 1–2 years.
- First-wave reforms were effective in increasing health workers numbers, availability and pay. More stubborn challenges remain however to be tackled, now intensified by the devastation of the Ebola epidemic. These include more effective packages to promote rural service; more effective recruitment, posting and management systems; and measures to ensure good quality of care.
- Sustaining the momentum for reform and also the financing of the increased financial commitments (increased salaries, rural allowances and PBF, amongst others) remains a challenge for the government of Sierra Leone.

## Introduction

Health worker recruitment, retention, distribution and performance are arguably the most critical factors affecting the performance of a health system. In post-conflict settings, where health systems and health worker livelihoods have been disrupted, the challenges facing the establishment of the right incentive environment are particularly important, and the contextual dynamics around them especially relevant to understand and incorporate sensitively into policy measures. Human resources development is an important part of rebuilding the health sector post-conflict but has received relatively little attention in the literature and may be overlooked by decision-makers and donors (Shuey *et al*. 2003; Pavignani and Colombo 2009; O’Hanlon and Budosan 2011).

A ReBUILD study aimed to fill that gap by documenting the evolution of incentives for health workers post-conflict and their effects^1^. The original research questions were not focused on the Free Health Care Initiative (FHCI) in Sierra Leone but the FHCI emerged as a key catalyst in a series of human resources for health (HRH) reforms (Bertone *et al*. 2014b). This article pulls together the findings relating to the FHCI with the objective of tracing how it changed the HRH landscape in Sierra Leone and the challenges which remain. The rationale for this is 2-fold: first, there is an acknowledged gap in the literature on the impact of fee exemption policies on health staff, and, conversely, the implications of staffing for fee exemption (McPake *et al*. 2013), which this study can contribute to filling. Second, the case of Sierra Leone is interesting, not least because, unlike in some other countries, the FHCI was approached not as a bolt-on, free standing programme, but as requiring a system-wide change. It is important to analyse how this affectedHRH, as a key health system building block, and what lessons can be drawn for countries adopting more systematic approaches to fee removal or reduction.

## Methods

### Study design

A retrospective and cross-sectional study utilizing both quantitative and qualitative methods was conducted. The timeframe for retrospective data collection was the period since the end of the conflict (2002) to the present day. Fieldwork was done between the end of 2012 and 2013.

### Study areas

Sierra Leone, with approximately 60 00 000 people, is divided into four regions (North, South, East and the Western Area). Each region is subdivided into districts and each district into chiefdoms. In total, there are 14 districts and 149 chiefdoms. Four districts were chosen to be representative of the different regions, urban/rural variations, remoteness/hard to reach areas and measures of poverty/need. The study sites were: Western Area (Urban/Rural), Kenema District (Eastern Region), Bonthe District (Southern Region) and Koinadugu District (Northern Region).

Bonthe and Koinadugu districts have very difficult terrains (riverine for Bonthe and mountainous for Koinadugu) and their population is among the most impoverished in Sierra Leone. Social amenities, electricity and piped water supply are lacking in Bonthe and Koinadugu. Thus, health workers are usually unwilling to work in these districts. Kenema and Western area have large urban and rural populations and referral hospitals.

### Research tools

Data were collected through the following methods: career histories with health workers, key informant interviews (KII), a health worker survey, document review and analysis of routine HRH data. These are described briefly in turn here. Findings are drawn from across these five tools.

1. Career histories of health workers

This research tool used a participatory approach involving drawing of life lines and in-depth interviews with purposively selected health workers (Wurie and Witter 2014b). An open topic guide was used, which covered the following topics:

- How and why they became health workers
- Their career path since they became health workers, and what affected during and after the conflict
- Their overall perception of their career in terms of motivating and demotivating factors before, during and after the conflict
- Challenges they face in their job and how they cope with them before, during and after the conflict
- Their career aspirations
- Their knowledge and perceptions of incentive policies during and after the conflict
- Recommendation for an effective retaining package for health workers in rural areas

A total of 23 in-depth interviews were conducted (Table 1). All of them had worked in the health sector since 2000.

**Table 1:** Career history sample, Sierra Leone health workers

	Number of respondents
By district	
Western area	11
Koinadugu	5
Kenema	4
Bonthe	3
By gender	
Females	12
Males	11
By cadre	
Community health workers/ CHOs	8
Nurses	5
Mid-wives	7
MOs	3
By facility type	
Primary	7
Secondary	4
Tertiary	12
Total	23

The data was analysed from verbatim transcripts using the thematic framework approach, with the following stages: transcribing the interviews, familiarization of the transcripts and the audio recordings, producing a coding framework, coding and identifying key themes from individual transcripts, merging themes, searching for key findings under each theme, comparing and finding associations, and providing explanations for the findings. The coding and analysis was led by a Sierra Leonean researcher, with cross-checking and second reading by a UK-based researcher.

2. Key informant interviews

**Figure 1:**
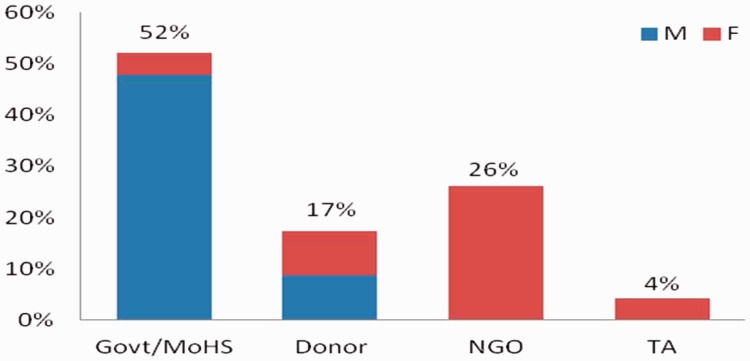
Summary of characteristics of key informants interviewed (ReBUILD KII, Sierra Leone)

A preliminary list of relevant key informants was drafted by the ReBUILD team in Sierra Leone (Bertone and Witter 2014). This list included both national and international organizations as well as individuals. Subsequently, a snow-balling technique was used to identify further informants. Twenty-three key informants were interviewed between October 2012 and June 2013. Most of the interviews (19) were carried out in Freetown, whilst 2 were conducted at district level. The remaining interviews (2) were done outside of Sierra Leone or by telephone. Twelve of the interviewees work or worked at the MoHS or with other governmental agencies. Six non-governmental organization representatives were interviewed, along with 4 donor representatives and 1 Technical Assistant (TA) (Figure 1). One researcher carried out the majority of the interviews and two additional researchers assisted them for five of the interviews.

A topic guide was prepared for use across all of the ReBUILD project countries and then it was adapted for use in Sierra Leone. The questions were sequenced in chronological order. Participants were asked about the HRH context in the immediate post-conflict period and the challenges that they faced. They were then asked about the policy responses to these challenges and what effects these had on the health system. Finally, they were asked to share any lessons learned from their experience and whether they had any recommendations for the future.

As described above, interviews were recorded and transcribed for thematic analysis. A UK-based researcher carried out the analysis and the other members of the team provided feedback on the initial results and on the draft of the report. An initial list of themes for the thematic analysis was drafted based on the findings of the document review (Table 3) and further themes were added based on the interview data analysis. As the same theme list was used, it was possible to triangulate the KII with information from the documentary review.

3. Health worker survey

The objective of the survey was to understand the incentive environment facing key kinds of health workers in Sierra Leone, their characteristics and the factors which motivate and demotivate them.

**Table 2:** Sampling frame of HWs by district (total number, original planned sample, actual sample)

Cadre	Western area			Koinadugu			Kenema			Bonthe			Total		
	Total staffing	Original sample	Actual sample	Total staff	Original sample	Actual sample	Total staff	Original sample	Actual sample	Total staff	Original sample	Actual sample	Total staff	Original sample	Actual sample
MO	24	12	7	3	2	2	4	2	0	4	2	2	35	18	11
Specialist doctors	12	6		1	1		2	1					15	8	
CHO/CHA	62	12	8	19	10	11	42	21	18	8	4	4	131	47	41
RN	138	14	10	13	7	6	21	11	7	5	3	2	177	33	25
SECHN	757	38	40	96	19	17	325	16	20	43	22	23	1221	95	100
EHO	85	17	6	9	5	2	11	6	4	6	3	2	111	30	14
MCH Aide	350	18	13	77	15	16	161	16	14	63	13	12	651	62	55
EDCU assistant	11	6	1	7	4	3	76	15	10	6	3	2	100	27	16
Lab technician	58	12	9	2	1	1	91	18	17	2	1	1	153	32	28
Pharmacy tech.	32	16	15	4	2	2	7	4	1	4	2	2	47	24	20
Other			1			0			0			1			2
Total	1529	150	110	231	64	60	740	109	91	141	52	51	2641	374	312

A structured questionnaire was used to collect data from all main cadres of health workers through face-to-face interviews (Witter *et al*. 2014a). The study population included Maternal and Child Health Aide (MCH Aide), State Enrolled Community Health Nurse (SECHN), Environmental Health Officer (EHO), Community Health Assistant (CHA), Community Health Officer (CHO), State Registered Nurse (SRN), mid-wives, pharmacists, laboratory technicians and doctors.

The sample size was based on the total number of workers in each category, with a smaller proportion chosen for larger groups. A planned total of 374 health workers (HWs) were included in the sampling frame (see Table 2). This constituted 14% of the estimated overall public workforce in these districts.

These cadres were identified from a range of facility types where they worked, to include rural and remote areas, as well as urban. They came from the public and private not-for-profit (PNFP) facilities. Sampling in selected facilities was pragmatic, but ensuring that the overall distribution of the sample reflected the sampling frame.

Actual numbers diverged somewhat from planned numbers in the different categories, largely due to limited number of staff of each category being found and available in the sites visited. The final sample was 312, instead of the planned 374. However, in relation to the total reported number of staff in the districts, this still constitutes nearly 12%, which is adequate. The main district which where it was hard to reach targeted numbers was Western Region.

The questionnaire focused on the current levels of income earned by health workers from different sources; work practices, including proportion of time spent by the worker in the public and private sectors; and willingness to work in rural or remote settings. In addition, the characteristics and practice of their main employment, including qualifications, years of work, regular workload and training, and earnings from both public and private sectors were included in the survey instrument. Furthermore, qualitative questions on motivating factors were incorporated into the questionnaire.

The quantitative data was coded, cleaned and analysed using Stata. Analysis was done by cadre, district, gender and public/PNFP employment status.

4. Document review

Documents were retrieved in 2012 by the ReBUILD team in Freetown, through contact with the MoHS, international donors and partners, other stakeholders and interviews with informants (Bertone *et al*. 2014a). A rapid internet search was also performed to identify articles in peer-reviewed journals and other relevant grey literature. A snowball technique was then adopted by which documents mentioned in other documents were actively searched from the source. If a theme or policy seemed under-represented, new searches were performed.

The initial search led to the identification of 76 documents. After an initial screening, 57 were deemed relevant for HRH issues in Sierra Leone and were fully reviewed. The documents dated from 2002 to 2012, with the vast majority from 2009 onwards. This may reflect the increased activity and investment levels in the sector post-2010 and/or the difficulty of retrieving earlier reports.

To analyse the documents collected, a series of ‘themes’ were identified and validated by the team. Themes and corresponding subthemes are listed in Table 3.

5. Secondary data analysis

A final research component involved analysis of routine staffing data collected by the MoHS (HRH directorate), with the objective of establishing changes in numbers, type, density, distribution, attrition, absenteeism and productivity over time (2002–12). Data were collected (Wurie and Witter 2014a) showing the:

- Changes in established posts for the different health professionals
- Changes in staffing numbers and types
- Changes in density of staff over the period
- Attrition, by cadre
- Absenteeism rates (only available 2010–14)

Gaps in output data (e.g. total number of outpatients, inpatients, facility deliveries, antenatal care (ANC) visits) at national level meant that productivity analysis was not possible, except for one district.

### Research ethics

Ethical approval was obtained from local and international research institutions in 2012. Informed consent was sought from the participants, assuring of confidentiality and anonymity of the information collected, and the research was undertaken in a sensitive manner, with data held securely.

## Results

### The context pre-FHCI

When Sierra Leone emerged from civil war in 2002, it faced human resource challenges common to post-conflict contexts—particularly, the absence of staff, who had fled, and the proliferation of NGO-supported services, with limited control by the MoHS overall. Gradually, during 2002–9, the MoHS re-established leadership, and a series of human resource policy documents and plans were produced, which presented clearly the challenges, but without having much traction, in terms of funding and momentum towards implementing the measures which they identified as needed. There were substantial gaps in posts filled and poor working conditions for staff, including low pay and difficulties getting on to payroll. Important disparities remained between salaries in the public sector and what can be earned outside of the civil service, particularly for the most skilled professionals. A comparison between the public and faith-based sector found 4- to 7-fold differences for senior medical officers (SMO) (Ensor* et al.* 2008). A large number of the people staffing public health facilities were ‘volunteers’—not on the payroll but maintaining themselves in large part through informal payments.

### Launch of FHCI

When the President announced the launch of the FHCI for pregnant and lactating mothers and children under five in November 2009, HRH care were picked out as an area needing immediate reinforcement as part of the policy’s implementation (Government of Sierra Leone 2009; Bertone *et al*. 2014b). In preparation for the FHCI launch, six technical working groups were put in place, one of which focused specifically on HRH issues. These groups held their meetings up to once a week during preparation phase (November 2009 to April 2010). They were tasked with designing the reforms and changes in the health system necessary to ensure the smooth roll out of the FHCI. They also co-ordinated different partners, assigned roles and identified available funding. Although there were disagreements within the group over priorities and the process was rushed, all sources agreed that the FHCI was the defining moment that shaped the healthcare system and gave a strategic approach to HRH policies.

### The main HRH reforms

The logic behind the HRH reforms was that if health care utilization was to increase then a number of chronic HR problems needed addressing, including:

- Fast-track recruitment and deployment to fill gaps in staffing
- Payroll cleaning to ensure that ‘ghost workers’ were taken off the payroll (and those who were working unpaid—the many ‘volunteers’—were added)
- Salary uplift to ensure that health workers were adequately paid and motivated to handle increased workload without imposing informal charges on users

This last reform was also influenced by a staff strike early in 2010, which was provoked in part by the feared consequences of the FHCI. These reforms were all introduced early in 2010 to prepare for the launch of the FHCI.

In a second round of HRH reforms, in 2011–12, a system of monitoring staff absences was introduced, linked to a new staff sanction framework. It aimed to ensure that the now more generously paid staff was actually at work. Another important policy introduced during this period was performance-based funding (PBF) to facilities. The PBF scheme aimed to meet the dual needs of providing some small flexible funding at facility level to replace lost user revenues (40% of PBF), as well as providing a direct incentive to staff (60% of PBF) to provide priority services. Finally, a remote allowance was introduced in January 2012 to encourage staff to take up postings in more rural, hard-to-serve areas.

## Implementation and effectiveness of reforms

The research tools provide details on the rationale, design, implementation and funding of these reforms, all of which were important to ‘protect the investment’ in FHCI. Broadly speaking, the first wave of reforms and the staff sanction framework were implemented effectively. The fast-track recruitment and deployment filled many gaps in staff, though it was a one-off process. Staff numbers doubled that year (Table 4), which represents a big increase on previous years’ trends, even allowing for the fact that some of these new recruits were already working but simply not on payroll.

**Table 3:** Themes and subthemes identified and used for thematic analysis (document review and KII)

Themes	Subthemes	
HRH context and challenges	Recruitment challenges	Changes to these challenges since 2002
	Distribution challenges	
	Retention challenges	
	Performance challenges (pay, motivation, management, etc.)	
Policy responses	Policy objectives and approaches	
	Drivers of change	
	Implementation of policies	
	Financing of policies	
	Impacts

**Table 4:** Changes in public health staffing numbers, 2005–11, Sierra Leone

	2005	2006	2007	2008	2009	2010	2011
MO/SMO	62	65	67	67	45	79	100
House officer (HO)	0	0	0	0	9	72	19
Registrar	8	8	8	6	6	5	4
Specialist/Senior specialist	12	12	12	12	12	40	47
Consultant	3	5	5	5	3	2	3
CHO	120	120	120	120	157	244	248
CHA	0	0	0	0	0	33	41
Senior registered nurse	227	227	227	274	190	223	271
Community health nurse	274	274	274	350	926	1354	1372
Mid-wife	70	70	70	70	78	60	47
Environmental officers	22	22	22	14	14	152	96
MCH aides	471	578	689	872	993	1892	1892
Endemic disease control staff	250	250	250	305	208	289	189
Lab technicians	18	18	15	150	21	128	85
Pharmacy	25	25	25	25	18	37	50
Pharmacy technician	250	22	22	222	43	146	211
Other	1205	1205	1345	1467	2040	4726	4672
Total	3017	2901	3151	3959	4763	9482	9347

Overall staff numbers tripled from 3017 in 2005 to 9482 in 2010. However, some key cadres were still very limited in terms of absolute numbers. Medical officers (MO) increased from 62 in 2005 to 100 in 2011, which is still very few for the whole country (50% of established posts). There has been a large increase in SECHNs (from 274 in 2005 to 1372 in 2011), but much less so for registered nurses (who only grew from 227 to 271 over the same period). Mid-wives actually dropped over the period, from 70 in 2005 to 47 in 2011). Disease control staff (tragically, in light of later events) dropped over the period from 250 to 189.

The payroll is now believed to be more robust, though it should be noted that more people were added than removed in the payroll cleaning. The new recruits included mainly those health workers who were previously working ‘voluntarily’ in the health facilities, remunerated on the basis of the internal facility revenues and informal payments from patients, but without receiving any compensation from the MoHS. Their support now shifted from community sources to the public (and donor) purse. These health workers were redeployed to the districts where needs were greater (Heywood 2010). A recent evaluation found that there had been ‘tremendous improvement’ in the quality of the MoHS payroll data management and that the cleaning had led to monetary savings of around $4 08 200 USD in the period between March 2010 and May 2012 (Stevenson *et al*. 2012).

**Figure 2:**
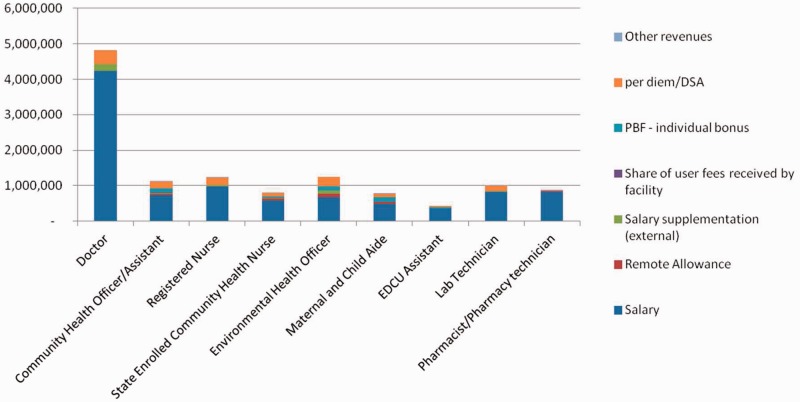
Breakdown of sources of primary income, by profession *Source:* Witter *et al.* 2014a.

Salary uplift has contributed to better motivation and retention, especially for higher level staff (the top grades seeing an increase of more than 700% in their salary, while for grade 3s it was 314%). The differential between doctors and other technical staff in current pay is shown in Figure 2. Salary is the main component, providing 88% of doctors’ primary income. In the survey, only 4% reported any revenues from user fees or any gifts from patients, which suggests that the FHCI is being relatively effectively implemented, though this finding needs cross-checking with patient reports.

**Figure 3:**
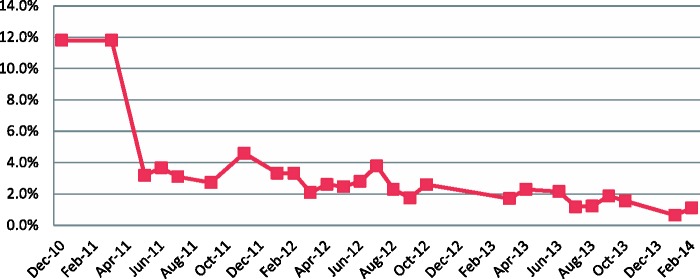
Rates of reported unauthorized absenteeism, Sierra Leone health workers, 2011–14 *Source:* payroll monitoring data, analysed in Wurie and Witter (2014a). *Note:* unauthorized absenteeism is calculated as ‘staff with one or more days of unauthorized absence /number of staff on payroll’.

Absenteeism is reported to have reduced and people have been sanctioned for non-attendance. Analysis of payroll monitoring data showed a significant drop from a baseline of 12% in December 2010, when the Staff Sanction Framework was implemented, down to 1.1% in February 2014 (Figure 3). However, two caveats remain for the analysis: the absence of baseline data prior to the FHCI or the framework’s introduction, and the need to continue with spot-checks to ensure that the reported data is robust.

Attrition, which is a combination of resignations, retirement, transfers and other factors, remained high over the period of 2005–11, but declined somewhat from 16% for all cadres in 2005 to 10% in 2010. However, there is considerable variation between cadres and by year (also reflecting the small numbers in some professional groups).

**Figure 4:**
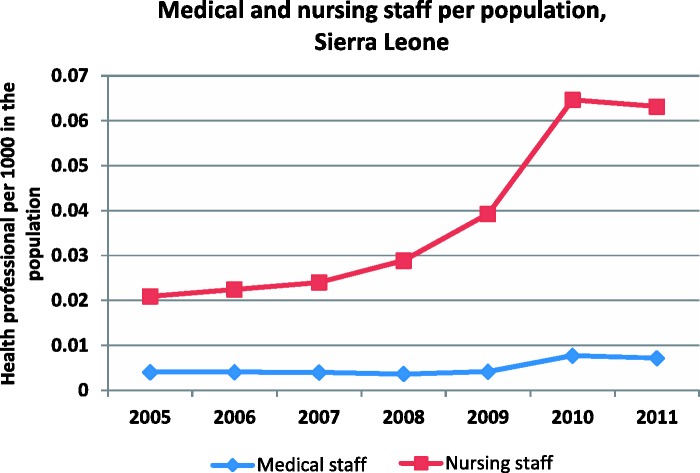
Density of medical and nursing staff in Sierra Leone (2005–11) *Source*: payroll data, analysed in Wurie and Witter (2014a). *Note:* For simplicity here, MO/SMO, HO, Registrars, Specialist/Senior Specialist, Consultant and CHO were classed as doctors. CHA, SRN, SECHN, Mid-wife, Environmental officers and MCH were classed as nurses.

In relation to population, the density of staffing remains low in Sierra Leone, but has improved over the past decade, and particularly around the period of introduction of the FHCI. In Figure 4, we have created a broad grouping of cadres into nursing and medical staff. Nursing staff to population increased gradually, accelerating in 2010 (65% increase that year, compared with 36% the year before). For medical staff, although the level is lower, the increase in ratios in 2010 was higher (86%, compared with 14% the year before).

In interpreting these figures, we need to bear two caveats in mind. First, the data may not be complete and comprehensive. Second, as people were taken on to payroll in 2010 who were already working as volunteers, some of the apparent increase that year was due to changes to the payroll rather than changes to actual numbers serving in the facilities. If during the payroll cleaning, 1000 added to payroll (Heywood 2010), then roughly 20% of the new staff were volunteers who were added rather than new recruits.

**Figure 5:**
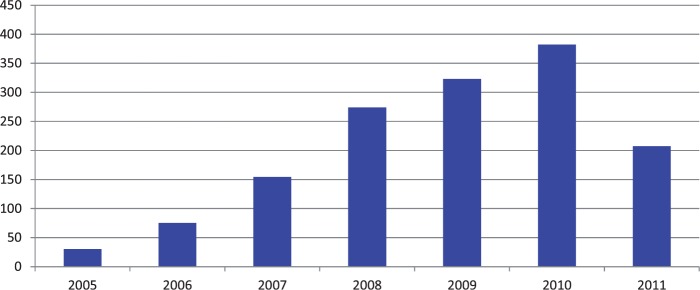
Change in combined institutional deliveries and ANC: staff ratio, Koinadugu district, 2005–11 *Note:* Institutional delivery calculated as having a 10:1 weighting compared with ANC visits; staff included here were all nurses and mid-wives (Wurie and Witter 2014a).

While gaps in the health management information system did not allow for output ratios to be calculated for health staff over the period of reform, data from individual study districts indicated, as expected, that the combined output to staff ratio increased. Although staff numbers increased, utilization of services increased more than proportionately in 2010, falling back in 2011 but still to a higher level than previously (Figure 5). The FHCI appears to have contributed to a longer term trend in increased health worker productivity.

While the first wave of reforms seems to have achieved a degree of success, the later reforms were apparently less effective in their implementation. Monitoring, feedback and payments under the PBF scheme are erratic and it remains poorly understood, though staff welcomes it if it can be strengthened. Of those surveyed by ReBUILD at primary health unit level, a third had received no PBF payment over the previous seven quarters (payments are meant to be quarterly), while others had only received from one to three payments, with delays. For the rural allowances, these are even more erratic and opaque, partly linked to funding problems.

In interviews, staff highlights benefits to themselves, in terms of pay and working conditions, as well as to the health system, in terms of increased use by patients and more investment in the services and facilities.

*‘… I was at [named facility] when it started and the influx of patients I saw, it was overwhelming. I was so happy that people who were afraid in the former days to come to the hospital because maybe they were not having money, they think they will be charged and so on, are coming in hundreds. … [..] … .If there was no free health, all these children who are so ill looking, these children who are so sick, these parents who look so poor, would die somewhere someday, not getting any care  … .[ … ] … The antenatal clinic sometimes you would go there you would have 4/5 patients for the day, now you have up to a 150 patients in one day coming there and they prefer coming here because they get drugs. If they are admitted, they are admitted, no admission fee, no bed fee and you have 3 meals a days’ (Female, Western Area, IDI-14)*

With regards to negative effects, the perceived increased workload was the most cited. Limited human resources means health workers are over stretched, especially in the provinces, work very long hours and have to be available 24 h. This sometimes leads to complications with care as some health facilities cannot cope effectively with the number of patients coming through.

‘Because we are working 24 hours and we are not, it’s not like a hospital were you have routine doctors, we are the only higher cadre personnel that work 24 hours so the work is strenuous. Before this time people are not coming because of finance but now once they remove the users’ fees people are coming 24 hours’ (Male, Koinadugu, IDI-11)

They also highlight the strains, e.g. of managing with too few staff, and perceive some negative effects, such as patients visiting repeatedly to seek free drugs and, for themselves, of having less time to pursue other activities, like private business. In relation to financial incentives, salaries are clearly the most important and reliable element, and the recent substantial pay increase is appreciated, though there is still a sense that it is not adequate in relation to the cost of living. Other allowances are seen as poor in terms of reliability and regularity.

### Conditions for success

Presidential support for the FHCI was recognized by all as critical to its success. The fact that donors were able to co-ordinate to support the FHCI was also of the highest importance. This also brought in a large number of short-term TAs, who played a role in enabling quick reforms in time for the launch. All of these factors remain important and are risks in relation to sustainability. For the first 3 years of funding the salary uplift, e.g. DFID paid 22% of the costs and the Global Fund 20%. For the PBF scheme, the World Bank is the funder, while the Remote Allowance is paid by the Global Fund.

### Unfinished agenda

Some reforms which are recognized to be important and which were planned for in the Free Care launch and the National Health Sector Strategic Plan (MoHS 2009) are still outstanding, perhaps because they require more institutional and deep-rooted reforms. Most sources agree that recruitment and deployment are too slow and centralized and that HR management should be devolved to district level. Within the Ministry, better co-ordination of HR policies is needed, avoiding ‘silos’ managed by different directorates. The new Health Service Commission is yet to be functional, and the performance management contracts for higher level managers are not fully operational. Measures to encourage and retain staff in rural areas require comprehensive packages, including housing and promotion and training opportunities. Revised training and measures focused on boosting quality of care are all part of the unfinished agenda.

There are still too few of some key cadres, such as mid-wives, and attrition remains high (13% in 2011, across all cadres). Self-reported working hours average 54 h per week across the staff surveyed by ReBUILD, who report seeing an average of 117 patients per week, which is relatively high (though for some categories these include preventive activities such as antenatal care and immunization, which can be less time-intensive). Questions on remuneration reveal substantial differences between doctors and the rest of the staff, with salaries of doctors more than four times that of registered nurses (a differential which increases when other sources are added). This may require attention, particularly given the low number of registered nurses and mid-wives.

Concentration curves and indices for health workers in the public, private and NGO sectors confirm the skewed distribution, with the exception of the NGO sector where staff distribution appeared to be more equal, reflecting the strong rural presence of NGOs (McPake *et al*. 2013). As a consequence, the availability of higher level professional health cadres outside of the Western Area is extremely low and the health system has to rely on MCH aides for the delivery of reproductive, maternal and newborn healthcare.

## Discussion

The story of the FHCI in Sierra Leone and its impact on HRH is an interesting one, which contrasts with the wider literature on user fee removal and human resources. Most studies to date in other countries point to a variety of issues which have arisen, including lack of consultation with staff about fee removal policies, lack of compensation to staff for the increase in workload which they usually face and general lack of linkage between health financing (user fee) and HRH policy-making (Witter *et al*. 2007; McPake *et al*. 2013). Evaluations have generally found staff supportive of the fee removal policies in principle, while also resentful in some cases of being ‘taken for granted’ by users who now expect all services to be fully free. A realist evaluation in four countries examined the way in which staff and managers adapt or adopt fee removal policies, dependent on their context and room for manoeuvre (Witter *et al*. 2014b).

Sierra Leone started from a difficult position, when the FHCI was announced in 2009. The health system was still weak from the war which ended only in 2002, and most of the basic building blocks, such as adequate staffing, were absent. Perhaps because of this weaker starting position, a much more holistic approach was adopted, which saw the need to address critical weaknesses in the health system pillars prior to the launch of the FHCI in April 2010. A combination of political momentum, donor buy-in and deployment of rapid technical assistance worked to bring in a series of major changes for health staff (Bertone *et al*. 2014b), which were not just one-off but continued to be rolled out over 2010–12. The closest parallel may be the abolition of user fees in Uganda in 2001, when staff salaries were raised in tandem and drug supply systems improved (Nabyongo *et al*. 2008). However, the FHCI in Sierra Leone led to an even more systematic attempt to address health system barriers, including changes to facility financing, procurement of pharmaceuticals, health information systems and governance, which included innovative use of civil society monitors at facility level. This was not always welcomed by staff, who commented in in-depth interviews on the difficulty of being monitored by untrained community workers.

This does not mean that the FHCI was able to address all HRH (or other health system) challenges effectively. It was introduced at speed and reforms were prioritized. Removing ‘ghost workers’ and conducting a rapid recruitment exercise, raising salaries and bringing in a system to ensure that staff were actually at work were the top priorities, and the evidence suggests that these were done with some effectiveness. Providing small flexible resources and incentives to focus on essential services at the primary level, as well as support for those working in rural areas, came next. At the time of the research, these policies were less effective, with payment to health workers for the PBF and remote allowance schemes limited, erratic and poorly understood by the health staff themselves. More generally, there was a sense of the wave of reforms stalling, with some more long-standing issues, such as improving and decentralizing the recruitment, deployment and management of HRH still unresolved. This article also highlights ongoing challenges (ones which predated the FHCI and still remain to be addressed), such as lack of certain cadres, unequal distribution of staffing across districts, the need for a revised training policy, and for a more systematic package of financial and non-financial incentives, especially for those working in rural areas.

These have been brought into sharp relief by the current Ebola epidemic, in which health workers have been amongst the main victims (Witter and Wurie 2014). Not only have many health workers been infected and killed by disease, they have also been stigmatized by communities as vectors of transmission, and many have left work and training (training centres have been closed, which will affect rebuilding the workforce). Although the epidemic has been poorly managed, it is arguable that the situation would have been far worse if there had been fewer staff, in general and on payroll. However that may be, it is clear that Ebola has eroded many of the gains documented in this study and that the health system as a whole is going to require another round of reconstruction, which should be informed by the lessons learned from previous experiences.

The study faced a number of limitations. The reforms were brought in nationwide from 2010 onwards, with no baseline data and no control areas. In particular, for a number of variables (such as distribution and absenteeism), data and documents were absent, particularly for the pre-FHCI period. Amongst its other impacts, the FHCI helped to bring in better monitoring systems, which is helpful for the period since 2010 but limits judgements in relation to changes before and after the FHCI. Some areas which are harder to measure, such as measures of technical quality of care provided by health staff are lacking, both before and after the FHCI. Informal payments, which were known anecdotally to be a significant coping strategy before the FHCI, are thought to be reduced, but this will require more in-depth study to confirm.

The study’s strength is that it is able to triangulate information and opinions from official documents, key informants, routine data and health workers (using both survey and in-depth interviews). This gives a very well-rounded picture. Moreover, as the study was focused on changes to health workers’ incentives post-conflict in Sierra Leone (not specifically on the FHCI), it is able to set the changes brought about by the FHCI in context and also highlight the current situation and challenges faced by health staff. Its analysis will feed into a wider evaluation of the FHCI which is ongoing, which will assess effects on other health pillars and also integrate other important viewpoints, such as communities’ awareness and perceptions of the FHCI.

## Conclusions

The FHCI had a major effect on health workers in Sierra Leone, triggering a series of reforms which significantly changed their number, pay and attendance. It also increased their workload, though this has yet to be fully quantified given gaps in the health information system. Overall, reported motivation has improved, though there remain tensions between different cadres (higher level staff benefited more than lower level staff) as well as a demand for a more consistent package of financial and non-financial incentives, particularly in rural areas.

The health system remains weak, and long-standing needs for more decentralized recruitment and management, e.g. remain to be addressed. The momentum of the ‘big bang’ of FHCI, which brought together high-level political will and donor support (reconciling divergent agendas under the pressure of an agreed urgent priority) was effective for a period, but now risks slowing down.

The FHCI experience showed what can be achieved when user fee removal is tackled in a more wholesale way, identifying and addressing the health system ‘blocks’ which need to be functioning effectively for the reform to work. However, sustaining and extending the gains is the current challenge—not only in terms of the number of staff still needed, but also their distribution and ensuring that they are enabled to work effectively and to provide high quality care for all. Post-Ebola, these challenges will be even more critical.

## Notes

^1^ ReBUILD is a research consortium focussing on the reconstruction of health systems post-conflict. Its focal countries are Sierra Leone, Zimbabwe, Cambodia and Uganda.
